# Packaging Alters Fresh Chicken Characteristics and Volatile Profiles During Refrigerated Storage

**DOI:** 10.3390/foods14193284

**Published:** 2025-09-23

**Authors:** Savannah L. Douglas, Nina E. Gilmore, Ricardo J. Barrazueta-Cordero, Xenia M. Contreras, Jase J. Ball, Don R. Mulvaney, Soren P. Rodning, Jason T. Sawyer

**Affiliations:** 1 Department of Animal Sciences, Auburn University, Auburn, AL 36849, USA; sld0060@auburn.edu (S.L.D.); neg0027@auburn.edu (N.E.G.); rzb0143@auburn.edu (R.J.B.-C.); jase.ball@zoetis.com (J.J.B.); mulvadr@auburn.edu (D.R.M.); rodnisp@auburn.edu (S.P.R.); 2 Department of Poultry Sciences, Auburn University, Auburn, AL 36849, USA; xmm0001@auburn.edu

**Keywords:** chicken breast, shelf-life, volatile compounds

## Abstract

Vacuum-based packaging is less frequently applied to poultry at the retail level. Evaluating the impact of vacuum packaging on fresh poultry may elicit extensions to storage life and reduce spoilage for consumer products such as fresh chicken. Boneless–skinless chicken breasts (N = 315; 105/treatment) were packaged using polyvinyl chloride (PVC) overwrap, vacuum packaging (VP), or vacuum skin packaging (VSP) and stored in simulated retail conditions for 20 days. Packages of fresh chicken were measured for changes in surface color, odor, pH, microbial growth, lipid oxidation, and volatile compounds. Packaging treatment significantly influenced surface color, with VP samples appearing lighter (*p* < 0.0001) and less red (*p* < 0.0001) than chicken packaged using PVC. Odor scores increased as storage time increased (*p* < 0.0001), and VSP maintained the most favorable odor throughout the 20-day refrigerated display. Lipid oxidation was greatest in PVC (*p* = 0.0338) packages on day 5 and lowest for VSP chicken packages on day 15. Electronic nose (e-nose) analysis concluded that packaging can influence aldehydes (*p* = 0.0025), alkanes (*p* = 0.0143), and terpene (*p* = 0.0214), compounds which have been associated with off-odors. In addition, microbial counts increased during storage time across all packaging types (*p* < 0.0001) but did not exceed a spoilage threshold of 7-log CFU/g throughout the 20 days of storage. The results conclude that vacuum-based packaging methods, either VP or VSP, can improve storage duration meat quality characteristics during refrigerated storage, and volatile e-nose compounds may be linked to the development of off-odors.

## 1. Introduction

Fresh poultry is a globally popular protein source due to its meal versatility, nutritional value, and affordability [1]. However, fresh chicken can deteriorate rapidly, creating significant challenges in the industry to provide quality and safe products throughout the supply chain [2]. Spoilage in fresh meat is primarily driven by microbial growth and biochemical changes. These changes can result in undesirable sensory characteristics such as off-odors, discoloration, and changes in texture [3]. Food packaging systems are crucial to extending the shelf-life of a product by reducing oxygen exposure, inhibiting microbial growth, and maintaining physicochemical and sensory attributes of a product [4]. Throughout the retail sector in the USA, polyvinyl chloride (PVC) packaging film with an expanded polystyrene tray is common for fresh poultry. However, PVC packaging is constructed with limited barrier properties, causing an acceleration of spoilage compared to packaging methods that limit oxygen exposure such as vacuum packaging (VP) and vacuum skin packaging (VSP) [5].

Technological developments in meat and food packaging have afforded proteins such as beef, pork, and lamb greater storage time. Vacuum packaging is becoming a more prominent method for consumer products throughout the retail sector. Fresh beef and pork have recorded a greater increase in retail counter square footage when offered to consumers using vacuum packaging [6]. Vacuum packaging or VSP is the result of removing atmospheric gases from the packaging environment to reduce the growth of spoilage organisms and therefore leading to an extension of storage life for fresh meats [7]. When using vacuum packaging, inhibition of aerobic organisms can occur; however, additional organisms classified as anaerobic or facultative anaerobic bacteria can be present within the package. Specific to fresh poultry when placed in vacuum packaging, microbial organisms can generate organic compounds capable of producing odors and associated with meat spoilage [3].

Vacuum packaging has been investigated and used for proteins like beef, pork, lamb, and goat from fresh to cooked products. However, the use of vacuum packaging throughout poultry products has been limited due to the development of undesirable odors, often described as sour, sulfurous, or metallic when packaged in barrier-type methods and stored refrigerated for excessive durations [8]. Confinement odors of fresh poultry may dissipate once the package is opened, but these odors could reduce consumer acceptability at the time of opening, increasing the percentage of discarding of fresh poultry that could be considered wholesome [9]. Packaged poultry odors have been linked to the accumulation of volatile organic compounds generated from microbial growth and oxidative processes [3]. It is well documented throughout the literature that confinement odors have been reported when using vacuum packaging for fresh chicken, hence the lack of use throughout the industry in the USA [8]. Identifying the specific volatiles in fresh packaged chicken that are responsible for confinement odors is critical for improving packaging methods, consumer acceptance, and a reduction in food waste.

Technology such as the electronic nose (e-nose) can detect and classify complex mixtures of volatile compounds from meat and food samples [10]. This instrument mimics the human olfactory system, providing quantifiable results for odor profiles. When used with other indicators such as microbial counts, surface color, and pH, e-nose technology can improve early detection of spoilage [11]. To facilitate improvements in packaging options for fresh chicken and increase consumer acceptance, the current study aims to identify associated volatiles that may be linked to deterioration of fresh chicken in storage when using vacuum packaging. It is hypothesized that vacuum-based packaging such as VP or VSP can significantly alter spoilage indicators compared to current PVC packaging of fresh chicken.

## 2. Methodologies

### 2.1. Fresh Chicken

Boneless–skinless chicken breast was purchased from a commercial poultry processor (Wayne Farms, Union Springs, AL, USA) within 12 h of harvest and transported under refrigerated conditions to Auburn University (Auburn, AL, USA). Chicken breast fillets were stored (24 h) in refrigeration (Model LEH0630, Larkin, Stone Mountain, GA, USA) at 2 °C ± 1.5 °C until packaging treatments were applied. Storage temperatures for this study agree with Untied States Department of Agriculture Food Safety Inspection Service (USDA-FSIS) recommended industry requirements for fresh meat products in the USA [12]. Fresh chicken breast (272 kg) was received in 8 boxes. Each box was considered a block to account for potential variation, and from each block, chicken breasts were randomly allocated to one of three packaging treatments to ensure equal representation.

### 2.2. Packaging and Display Conditions

Film combinations for packaging treatment and compositions are displayed below (Table 1). Chicken breasts assigned to PVC were placed on a foam tray (2S, GENPAK, Charlotte, NC, USA) with an absorbance pad (DRI-LOC AC-50, Novipax, Oak Brook, IL, USA) and wrapped by hand. Vacuum packaging samples were packaged using a Variovac Optimus system (OL0924, Variovac, Zarrentin, Germany) and a Multivac system (KGD-87787 R175, Sepp Haggenmüller GmbH & Co., Wolfertschwenden, Germany) for vacuum skin packaging samples. Packaged chicken breasts were stored in a two-door, refrigerated lighted display case to simulate retail display (Model 178GDC49HCB, Clark Associates Inc. DBA Avantco Equipment, Lancaster, PA, USA) under continuous LED lighting with an intensity of 2297 lux for each shelf. Packaged chicken breasts were distributed across the refrigerated shelves and stored for 20 days at 2 °C ± 1.5 °C. Every 5 days throughout the storage duration, samples were evaluated for instrumental surface color, lipid oxidation, pH, volatile compounds, odor, and microbial count.

### 2.3. Instrumental Color

Each surface of the chicken breast fillet (n = 10/treatment) was scanned using a HunterLab MiniScan EZ colorimeter (Model 45/0 LAV, Hunter Associates Laboratory Inc., Reston, WV, USA) every 5 days for the duration of the study. Surface color readings were determined from the mean of three readings of each sample using illuminant D65 with a 10° observer and 31.88 mm aperture using the American Meat Science Association guidelines [13]. Prior to scanning, calibration of the colorimeter was achieved using the black and white tile provided by the manufacturer.

### 2.4. Odor Evaluation and pH Analysis

Packaged chicken breasts (n = 7/treatment/day) were opened, and within 5 s trained laboratory personnel evaluated the odor. Subjective odor scores were obtained by opening each package, smelling the sample, and recording a rating. Odor was rated on a 5-point scale adapted from American Meat Science Association guidelines [13]: 1 = fresh chicken, no off-odor; 2 = slight off-odor; 3 = noticeable off-odor; 4 = moderate off-odor, unacceptable; 5 = extremely unacceptable, extreme off-odor.

Postmortem pH of chicken breasts (n = 7/treatment/day) were measured by using a spear tip pH probe. A pH meter (Model-HI98163, Hanna Instruments, Woonsocket, RI, USA) was inserted into the geometric center of the breast fillet and a mean of three readings. Calibration of the pH meter was completed (pH 4.0 and pH 7.0) using 2-point standard buffers (Thermo Fisher Scientific, Chelmsford, MA, USA) prior to sampling.

### 2.5. Electronic Nose Analysis

Volatile compounds of raw chicken breast samples were analyzed by using an electronic nose (Heracles Neo e-nose, Alpha MOS, Toulouse, France) containing an autosampler. Volatiles were measured in triplicates (n = 2 chicken breast fillets/treatment). Samples were collected on days 0, 5, 10, 15, and 20. E-nose evaluation of compounds were measured using gas chromatography. Using a fume hood, chicken breasts from each treatment were minced by hand, and 2.00 g was weighed then placed into 20 mL vials. Vials were agitated at 500 rpm, and a 50.0 °C incubation temperature for 20 min was used to generate volatiles for headspace analysis. Following the incubation period, the autosampler injector inserted 5000 mL of headspace gas at 125 mL/s for odor concentration inside the trap. Trapping condition of each vial was maintained at 40 °C for 50 s. Hydrogen gas was used at a flow rate of 1 mL/min to carry the volatile components into the non-polar (MXT-5) and polar (MXT-1701) capillary columns for chromatographic analysis using a parallel, two-flame ionization detector (FID1 and FID2). Dual columns (180 mm in diameter) were used for gas chromatography with a length of 10 m. The final temperature of the analysis sequence was increased to 250 °C at 1 °C/s from 40 °C temperature according to described methods [14]. Peaks of the chromatogram were identified by comparing the retention time of each compound with the corresponding retention index. Detected compounds were evaluated using a relevance index calculated by ArochemBase Software (Alpha MOS, Toulouse, France, Version 4.7.0). Compounds with a relevance index greater than 70 were considered for analysis and interpretation.

### 2.6. Lipid Oxidation

Chicken breasts were analyzed for 2-thiobarbituric acid reactive substances (TBARS) (n = 7/treatment/day) using previously described methods [15]. Chicken breasts were minced by hand to provide a uniform sample, and 2.0 g of minced meat was homogenized for 45 s with 8 mL of 50 mM phosphate buffer containing 0.1% EDTA, 0.1% n-propyl gallate, and 2 mL of trichloroacetic acid (Sigma-Aldrich, Saint Louis, MO, USA). After homogenization, samples were filtered using Whatman No. 1 filter paper. Duplicate 2 mL portions of the clear filtrate were transferred into 10 mL borosilicate tubes, mixed with 2 mL of 0.02 M 2-thibobarbituric acid reagent (Signa-Aldrich, Saint Louis, MO, USA), and boiled for 20 min. After boiling, the tubes were placed into an ice water bath for 15 min. Absorbance was measured at 533 nm using a spectrophotometer (Turner Model-SM110245, Barnstead International, Dubuque, IA, USA) and multiplied using a factor of 12.21 to obtain the TBARS value (mg malonaldehyde/kg of meat).

### 2.7. Microbiological Analysis

Packaged samples of fresh chicken (n = 7/treatment/day) were transported using refrigeration to a contract laboratory for analysis (Food Safety Net Services, Tucker, GA, USA). Samples were collected every 5 days throughout the 20-day retail display period. Anaerobic plate counting (ANA) was determined in triplicate using internal laboratory methods. Reagents and media preparation were conducted according to standard laboratory procedures. Samples weighing 3.00 g were aseptically removed from each package and homogenized for 120 s with a stomacher (BagMixer 400W, Interscience, Saint Nom la Bretêche, France) on medium-high in buffered peptone water. Petri dishes containing 12–15 mL of molten standard methods agar (SMA) were tempered between 44 °C and 46 °C and added to each plate within 15 min of homogenate preparation. SMA solutions were mixed and allowed to solidify on each plate prior to dilution. Within 15 min of preparing the homogenate, 1 mL of final dilution was placed on each sterile petri dish. Petri plates were inverted and placed inside an anaerobic container with anaerobic generation sachets. Samples were incubated at 35 ± 2 °C for 48 ± 2 h before sample counting was completed by trained laboratory personnel.

Microbial analysis was conducted in triplicate on packaged chicken breast (n = 7/treatment/day) using aerobic (APC) and lactic acid (LAB) Petrifilm plate counting. A 1:10 dilution using 3.00 g of aseptically removed chicken and buffered peptone water was homogenized for 60 s using a stomacher (BagMixer 400W, Interscience, Saint Nom la Bretêche, France); after stomaching, 1 mL was removed from each sample bag and placed in the center of the assigned Petrifilm (Neogen Corporation, Lasing, MI, USA). Using a 3 M plastic Petrifilm spreader, the homogenate was distributed evenly over the entire surface. Petrifilm was dried for 1 min allowing the solidifying agent to gel. Plated samples were incubated aerobically at 35 ± 1 °C for 48 ± 3 h before plate counts were conducted.

### 2.8. Statistical Analysis

Data analysis was performed using the GLIMMIX procedures of SAS (version 9.2; SAS Inst., Cary, NC, USA). Computation of least square means was completed for all dependent variables (instrumental color, odor evaluation, pH, lipid oxidation, microbial growth, and volatile analysis). Fixed effects of packaging treatment, day, and their interaction were included in the model with sample serving as the experimental unit. Significant (*p* ≤ 0.05) means were separated using pairwise *t*-tests (PDIFF option).

## 3. Results and Discussion

### 3.1. Instrumental Surface Color

**Figure 1:**
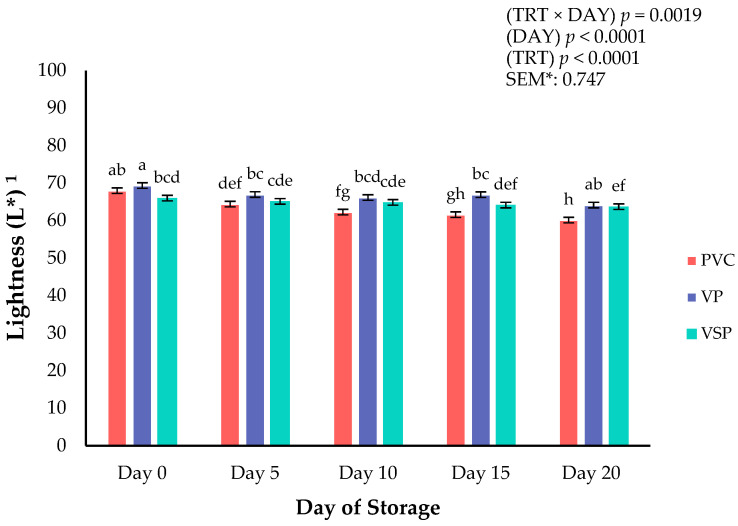
Interaction of packaging treatment × day of storage for surface lightness (L*) on boneless–skinless chicken breast. ^a–h^ Means lacking common superscripts differ (*p* < 0.05). * SEM, standard error of the mean. Lightness (L*) values are a measure of darkness to lightness where 100 is white, and 0 is black. Packaging treatment (N = 10/treatment): PVC—10 μm polyvinyl chloride. OTR: 14,000 mL/m/24 h. VTR: 30 g/m/24 h. VP—forming: 175 μm nylon/EVOH/enhanced polyethylene coextrusion. OTR: 0.4 ML/m/24 h. VTR: 3.3 g/m/24 h. VP—non-forming: 65 μm nylon/polyolefin plastomer coextrusion. OTR: 53.0 mL/m/24 h. VTR: 6.5 g/m/24 h. VSP—forming: 350 μm polyester/EVOH/polyethylene coextrusion. OTR: 1.8 mL/m/24 h. VTR: 2.8 g/m/24 h. VSP—non-forming: 127 μ PE/EVOH/LLDPE. OTR: 0.8 mL/m/24 h. VTR: 0.4 g/m/24 h.

**Figure 2:**
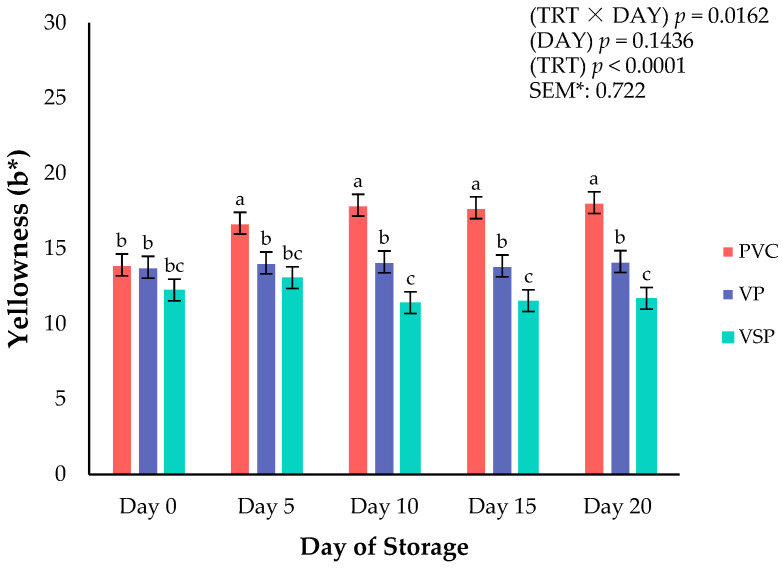
Interaction of packaging treatment × day of storage for surface yellowness (b*) on boneless–skinless chicken breast. ^a–c^ Means lacking common superscripts differ (*p* < 0.05). * SEM, standard error of the mean. Yellowness (b*)—a larger value indicates a more yellow color, where +60 is yellow and −60 is blue. Packaging treatment (N = 10/Treatment): PVC—10 μm polyvinyl chloride. OTR: 14,000 mL/m/24 h. VTR: 30 g/m/24 h. VP—forming: 175 μm nylon/EVOH/enhanced polyethylene coextrusion. OTR: 0.4 ML/m/24 h. VTR: 3.3 g/m/24 h. VP—non-forming: 65 μm nylon/polyolefin plastomer coextrusion. OTR: 53.0 mL/m/24 h. VTR: 6.5 g/m/24 h. VSP—forming: 350 μm polyester/EVOH/polyethylene coextrusion. OTR: 1.8 mL/m/24 h. VTR: 2.8 g/m/24 h. VSP—non-forming: 127 μm PE/EVOH/LLDPE. OTR: 0.8 mL/m/24 h. VTR: 0.4 g/m/24 h.

**Figure 3:**
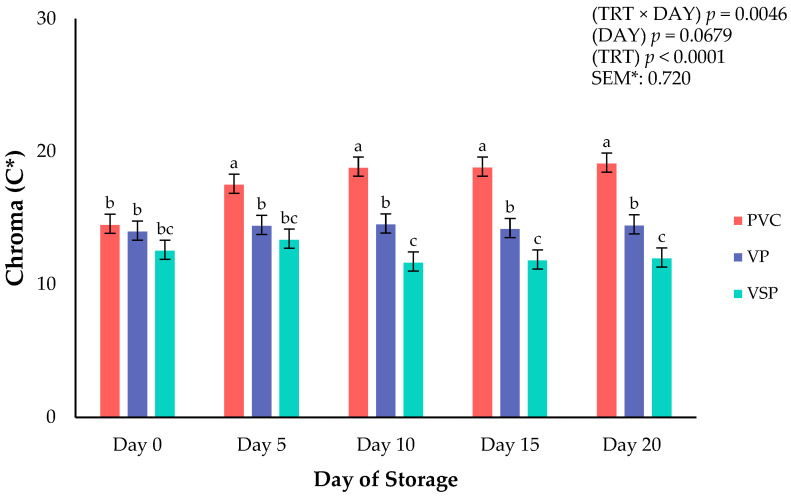
Interaction of packaging treatment × day of storage for chroma (C*) of boneless–skinless chicken breast. ^a–c^ Means lacking common superscripts differ (*p* < 0.05). * SEM, standard error of the mean. Chroma (C*)—total color (a larger number indicates a more vivid color). Packaging treatment (N = 10/Treatment): PVC—10 μm polyvinyl chloride. OTR: 14,000 mL/m/24 h. VTR: 30 g/m/24 h. VP—forming: 175 μ nylon/EVOH/enhanced polyethylene coextrusion. OTR: 0.4 ML/m/24 h. VTR: 3.3 g/m/24 h. VP—non-forming: 65 μm nylon/polyolefin plastomer coextrusion. OTR: 53.0 mL/m/24 h. VTR: 6.5 g/m/24 h. VSP—forming: 350 μm polyester/EVOH/polyethylene coextrusion. OTR: 1.8 mL/m/24 h. VTR: 2.8 g/m/24 h. VSP—non-forming: 127 μm PE/EVOH/LLDPE. OTR: 0.8 mL/m/24 h. VTR: 0.4 g/m/24 h.

Surface color is a critical attribute that influences consumer purchasing decisions of all fresh meats at the retail counter. Consumers weigh the visual indicators when shopping at the meat counter to assess the fresh and wholesome quality of a meat product [16,17]. Instrumental surface color was recorded on boneless–skinless chicken breasts every 5 days throughout a 20-day refrigerated storage period. Significant interactions of packaging treatment × day of storage were observed for lightness (*p* = 0.0019; Figure 1), yellowness (*p* = 0.0162; Figure 2), and chroma (*p* = 0.0046; Figure 3). These interactions conclude that packaging was instrumental to the surface color changes throughout the storage duration. Specifically, VP and VSP samples were lighter (L*) from day 10 to the end of the storage period than chicken packaged in PVC. It appears that vacuum packaging can provide fresh chicken with a surface color that is more stable for lightness and chroma (vividness) because of reduced oxygen exposure. Packaging treatment (Table 2) influenced all surface color parameters except for hue angle (*p* = 0.1749). It has been reported that absence of oxygen in vacuum packaging conditions can reduce the formation of oxymyoglobin, leading to a lighter appearance [17]. Regardless, PVC packaging is superior for affording greater oxygen permeability, allowing for a redder product and more vivid (greater chroma values) surface color. These results align with previous findings that high-oxygen environments enhance surface color but could lead to increased oxidation and discoloration over time [16]. Overall, the packaging environment is important in regulating color changes and degradation over time. Earlier studies suggest a trade-off between visual appeal and shelf-life under various packaging environments, which is supported in the current results [18,19,20].

In addition to packaging type, storage time was also analyzed for changes in surface color (Table 3). Storage time significantly affected lightness (*p* < 0.0001) and redness (*p* = 0.0100) throughout the duration of the study. Lightness values decreased over time, indicating the surface color became darker as storage duration increased. This could be associated with protein denaturation, oxidation, or microbial growth [20]. No significant effect of storage time was observed for hue angle or chroma, which may suggest stability in the perceived color during storage despite changes in lightness and redness.

### 3.2. Odor Evaluation and pH

**Figure 4:**
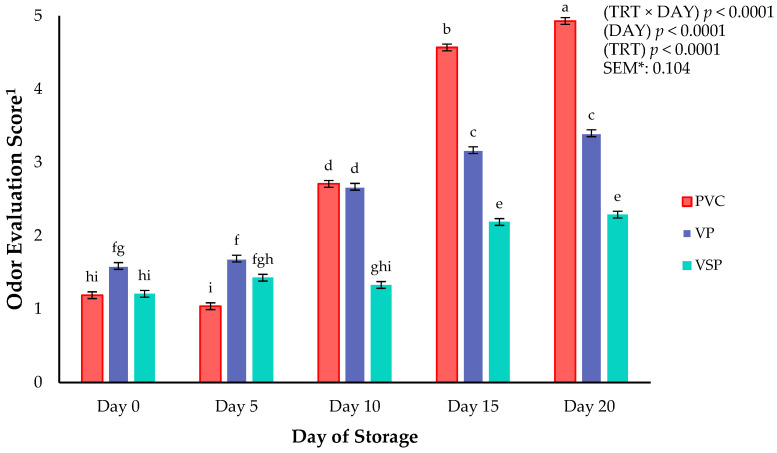
Interaction of packaging treatment × day of storage on odor evaluation scores of boneless–skinless chicken breast. ^a–i^ Means lacking common superscripts differ (*p* < 0.05). * SEM, standard error of the mean. ^1^ Odor evaluation score anchors: 1 = “fresh chicken, no off odor”; 2 = “slight off odor”; 3 = “small off odor”; 4 = “moderate off odor, unacceptable”; 5 = “extremely unacceptable, extreme off odor”. Packaging treatment (N = 7/Treatment): PVC—10 μm polyvinyl chloride. OTR: 14,000 mL/m/24 h. VTR: 30 g/m/24 h. VP—forming: 175 μm nylon/EVOH/enhanced polyethylene coextrusion. OTR: 0.4 ML/m/24 h. VTR: 3.3 g/m/24 h. VP—non-forming: 65 μm nylon/polyolefin plastomer coextrusion. OTR: 53.0 mL/m/24 h. VTR: 6.5 g/m/24 h. VSP—forming: 350 μm polyester/EVOH/polyethylene coextrusion. OTR: 1.8 mL/m/24 h. VTR: 2.8 g/m/24 h. VSP—non-forming: 127 μm PE/EVOH/LLDPE. OTR: 0.8 mL/m/24 h. VTR: 0.4 g/m/24 h.

Odor directly impacts consumer acceptability and perceived quality of a product. [21]. In the current study, there was a significant packaging treatment × storage day interaction for odor evaluation scores (*p* < 0.0001) (Figure 4), indicating that changes in odor scores over time are dependent on packaging type. Odor scores for PVC-overwrapped samples increased rapidly after day 10, reaching the highest values by days 15 and 20. Vacuum packaging also showed an increase in odor scores over storage, but values remained lower than PVC at later timepoints. In contrast, vacuum-skin-packaged samples exhibited the most stable odor profile, with minimal changes throughout day 10 and only a slight increase through the rest of the study. However, VSP samples’ scores remained lower than PVC and VP on day 20. Across all packaging types, odor scores increased steadily from day 0 until day 20, confirming that odor deterioration was progressive with storage duration. The results of the current study confirm theories regarding oxygen transmission rates (OTRs) of packaging films. As the OTR increases for packages of fresh chicken using PVC, the volume of atmospheric gases that pass into and out of the package is much greater than VSP or VP. Therefore, odor scores for PVC packages in the current study are minimal due to the OTR of the packaging film. Previous research conducted by this lab has concluded that vacuum skin packaging of chicken breast and thighs maintained a favorable odor throughout storage [22]. The results indicated VSP is superior in controlling odor of fresh chicken to VP and PVC overwrap due to the tight film-to-meat contact and limited oxygen transmission rate [22]. Since VSP and VP provide a lower oxygen transmission rate, this likely limits microbial growth and oxidation, which delays the development of off-odors [23]. However, even with these properties, odor scores still increased with storage duration. An increase in odor evaluation scores between day 10 and day 15 could be linked to the accumulation of amines, sulfides, and other volatiles associated with protein degradation [24].

**Figure 5:**
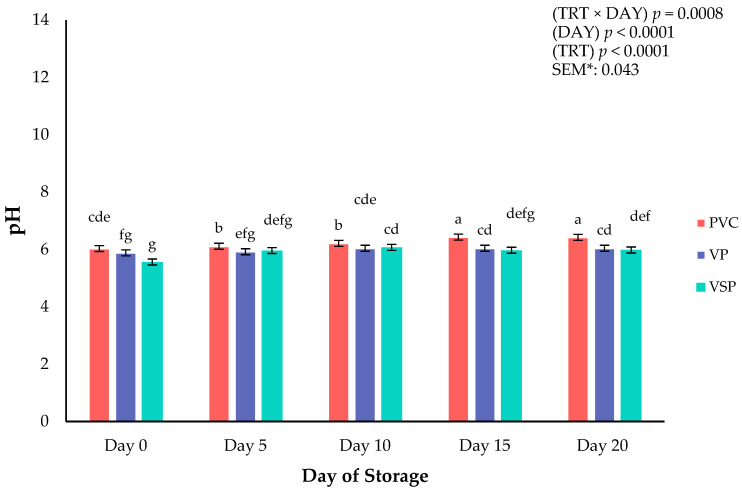
Interaction of storage packaging treatment × day of storage on pH of boneless–skinless chicken breast. ^a–g^ Means lacking common superscripts differ (*p* < 0.05). * SEM, standard error of the mean. Packaging treatment (N = 7/Treatment/Day): PVC—10 μm polyvinyl chloride. OTR: 14,000 mL/m/24 h. VTR: 30 g/m/24 h. VP—forming: 175 μ nylon/EVOH/enhanced polyethylene coextrusion. OTR: 0.4 ML/m/24 h. VTR: 3.3 g/m/24 h. VP—non-forming: 65 μm nylon/polyolefin plastomer coextrusion. OTR: 53.0 mL/m/24 h. VTR: 6.5 g/m/24 h. VSP—forming: 350 μm polyester/EVOH/polyethylene coextrusion. OTR: 1.8 mL/m/24 h. VTR: 2.8 g/m/24 h. VSP—non-forming: 127 μm PE/EVOH/LLDPE. OTR: 0.8 mL/m/24 h. VTR: 0.4 g/m/24 h.

The postmortem pH of meat was recorded and can reflect chemical changes that occur during refrigerated storage. In addition to chemical changes, pH can lead to altered surface color of fresh meat [25], with a combination of changes in color, moisture, and texture occurring because of variation in postmortem pH in meat proteins such as fresh chicken [24,25,26]. Variation in muscle pH can also be an indicator of spoilage and microbial growth that occurs during storage conditions [26]. Packaging treatment × storage day interaction (Figure 5) had a significant effect on the pH in the current study (*p* = 0.0008). Across all days, PVC samples had higher pH values compared with VP and VSP. The pH increased during storage for all treatments until day 15, when values plateaued. Greater pH values in PVC are likely due to the high oxygen transmission rate, which permits increased microbial growth and oxidative processes [27]. In contrast, VP and VSP restrict oxygen exposure, slowing microbial growth and maintaining lower pH values. With an increase in pH values, the results indicate ongoing spoilage processes throughout all treatment types. The current results align with previous studies supporting theories that low-oxygen packaging options can delay biochemical changes but not fully prevent changes in poultry meat during storage [28].

### 3.3. Volatile Compounds

Overall, the greatest influence on fresh chicken storage-related changes were observed in sulfur and alkane compounds, suggesting that these compounds are contributing to poultry confinement odors. Further research on these specific volatiles could help develop storage life monitoring and packaging strategies for fresh chicken to mitigate the development of spoilage odors. Volatile compound development is a key indicator of meat spoilage that can contribute directly to odor and overall sensory quality [29]. These compounds are often formed through chemical and microbial processes during storage [27,29]. An electronic nose (e-nose) was used to identify volatiles contributing to odor development in vacuum-packaged fresh chicken. The intensity of these volatiles was affected by packaging type throughout the storage duration. The main effects of packaging significantly increased the peak area for several e-nose volatile compound groups, including aldehydes (*p* = 0.0025), alkanes (*p* = 0.0143), ketones (*p* = 0.0302), esters (*p* = 0.0227), and terpenes (*p* = 0.0003) presented below (Table 4). Hydrocarbons were the most abundant group across all packaging treatments, with the greatest values observed in PVC and VP samples. Hydrocarbon peak areas were reduced in VSP samples, likely due to the lower oxygen transmission rate and the rigidity of the skin-packaging application. Chicken breasts packaged using VP and PVC have greater flexibility within the package for atmospheric gases to be trapped and utilized by the proteins during storage. Despite greater peak area values, hydrocarbons have not been associated with the development of off-odors in fresh meats [30].

Storage duration (day) significantly influenced the formation of alkanes (*p* = 0.0118) and sulfur compounds (*p* = 0.0040), whereas alcohols (*p* = 0.3167), terpenes (*p* = 0.0941), ketones (*p* = 0.3198), and esters (*p* = 0.8118) were not affected (Table 5). Alkane concentrations increased throughout the 20-day simulated storage period. Sulfur-containing compounds varied significantly (*p* = 0.0440) with storage duration (Table 5); more specifically, a marked increase in sulfur compounds occurred after day 10. Increased sulfur compounds have been linked to greater microbial activity from anaerobic bacteria that generate sulfurous volatiles such as hydrogen sulfide and dimethyl sulfide [3]. Dimethyl sulfide was detected in all samples, regardless of packaging treatment or storage day. Low-oxygen environments often favor the growth of spoilage bacteria that degrade sulfur-containing amino acids into volatile sulfur compounds, including dimethyl sulfide, which has been characterized by cabbage-like or sulfurous odors [31]. It appears the quantities of dimethyl sulfide are responsible for the off-odors associated with poultry spoilage of vacuum-packaged chicken. Similar results for vacuum-packaged turkey reported dimethyl sulfide, dimethyl disulfide, and dimethyl trisulfide as the primary contributors to irradiation-related off-odors [32]. In addition to dimethyl disulfide or trisulfide, elevated sulfur volatiles have been widely recognized as indicators of protein degradation and spoilage [3].

Retention time, relative index, and sensory attributes associated with volatile compounds from e-nose detection are presented below (Table 6 and Table 7). Surprisingly, compounds (alkane and sulfur) which are often associated with confinement odors of fresh chicken (meaty and putrid) when stored using vacuum packaging were prevalent. Alkanes, sulfur, furan, hydrocarbons, and aldehydes had the highest relevance indices across all storage days and packaging treatments. These trends for volatile compounds further support previous results suggesting that perceived freshness of poultry is linked to sulfur and hydrocarbon chemistry throughout postmortem refrigerated storage [32,33]. Additional research is needed to further elicit the time during storage when these meat characteristics occur along with the flavor decline when extending storage of fresh chicken.

### 3.4. Lipid Oxidation

**Figure 6:**
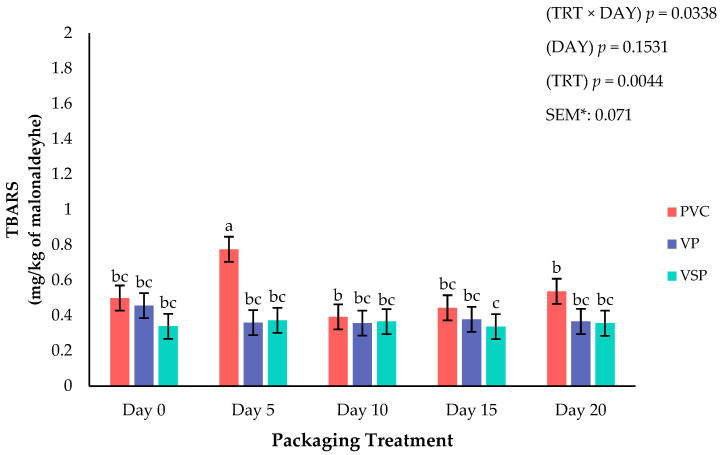
Interaction of packaging treatment × day of storage on lipid oxidation (TBARS). ^a–c^ Means lacking common superscripts differ (*p* < 0.05). * SEM, standard error of the mean. Packaging treatment (N = 7/Treatment/Day): PVC—10 μm polyvinyl chloride. OTR: 14,000 mL/m/24 h. VTR: 30 g/m/24 h. VP—forming: 175 μm nylon/EVOH/enhanced polyethylene coextrusion. OTR: 0.4 ML/m/24 h. VTR: 3.3 g/m/24 h. VP—non-forming: 65 μm nylon/polyolefin plastomer coextrusion. OTR: 53.0 mL/m/24 h. VTR: 6.5 g/m/24 h. VSP—forming: 350 μm polyester/EVOH/polyethylene coextrusion. OTR: 1.8 mL/m/24 h. VTR: 2.8 g/m/24 h. VSP—non-forming: 127 μm PE/EVOH/LLDPE. OTR: 0.8 mL/m/24 h. VTR: 0.4 g/m/24 h.

TBARS can be a major influence on the quality, shelf-life, and consumer acceptance of poultry meat [32,34]. Chicken breast can be susceptible to oxidative deterioration due to its greater proportion of polyunsaturated fatty acids [33]. Throughout the literature, it has been confirmed that meats containing greater fat will need protection in the form of packaging or antioxidant ingredients from the detrimental effects of lipid deterioration. Specifically, storing fresh meats can create oxidative spoilage and the development of off-flavors, odors, and discoloration, which negatively impacts product marketability [35]. As the presence of oxygen plays a key role in accelerating lipid oxidation, packaging methods such as vacuum or VSP that limit oxygen exposure have been widely studied and utilized as a method to preserve meat quality for proteins like beef, pork, or lamb [35]. Studies have concluded that vacuum packaging and vacuum skin packaging are effective in limiting oxygen transmission and improving the storage of meat products [36]. There was an interaction (*p* = 0.0338) of packaging treatment × storage day for TBARS on fresh chicken. In addition, fixed effects for TBARS were significantly affected (*p* = 0.0044) by packaging treatment, and PVC packages displayed a greater amount of oxidation when compared to vacuum packaging and vacuum skin packaging (Figure 6). Current results are consistent with previous reports suggesting that oxygen-permeable films such as PVC accelerate oxidation due to the increase oxygen availability [19]. Additionally, the use of vacuum packaging resulted in lower TBARS, indicating slower oxidative deterioration.

**Figure 7:**
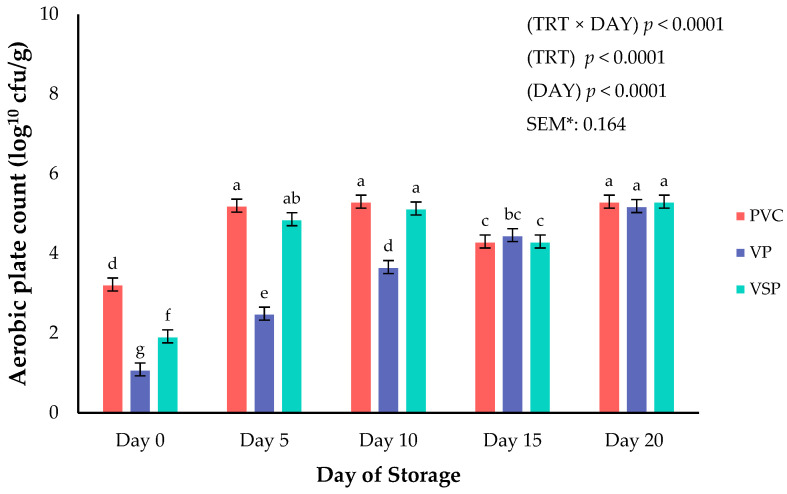
Interaction of packaging treatment × day of storage on aerobic plate count. ^a–g^ Means lacking common superscripts differ (*p* < 0.05). * SEM, standard error of the mean. Packaging treatment (N = 7/Treatment/Day): PVC—10 μm polyvinyl chloride. OTR: 14,000 mL/m/24 h. VTR: 30 g/m/24 h. VP—forming: 175 μm nylon/EVOH/enhanced polyethylene coextrusion. OTR: 0.4 ML/m/24 h. VTR: 3.3 g/m/24 h. VP—non-forming: 65 μm nylon/polyolefin plastomer coextrusion. OTR: 53.0 mL/m/24 h. VTR: 6.5 g/m/24 h. VSP—forming: 350 μm polyester/EVOH/polyethylene coextrusion. OTR: 1.8 mL/m/24 h. VTR: 2.8 g/m/24 h. VSP—non-forming: 127 μm PE/EVOH/LLDPE. OTR: 0.8 mL/m/24 h. VTR: 0.4 g/m/24 h.

Interestingly, there was no significant effect for day of storage (Figure 7) on TBARS (*p* = 0.1531). This lack of impact across day of storage suggests lipid oxidation remained relatively stable throughout the 20-day storage period, further supporting the results of packaging TBARS that conclude, when oxygen exposure is minimized, oxidative degradation can be delayed during storage.

### 3.5. Microbial Growth

**Figure 8:**
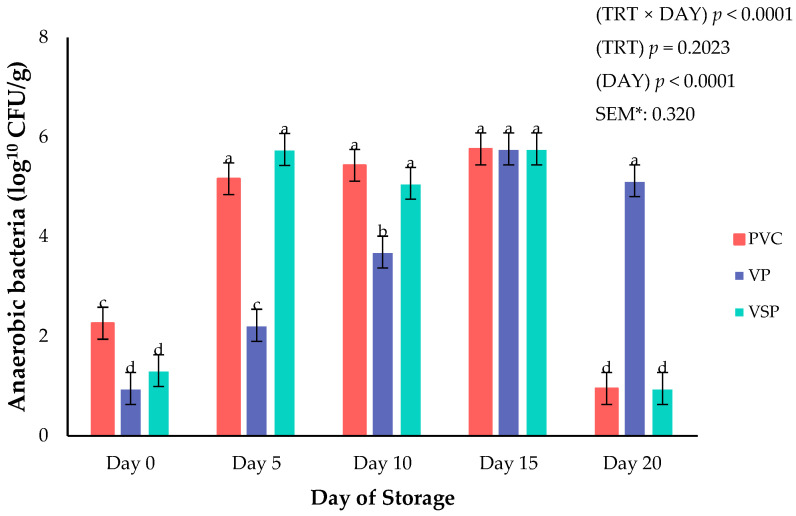
Interaction of packaging treatment × day of storage on anaerobic bacteria growth. ^a–d^ Means lacking common superscripts differ (*p* < 0.05). * SEM, standard error of the mean. Packaging treatment (N = 7/Treatment/Day): PVC—10 μm polyvinyl chloride. OTR: 14,000 mL/m/24 h. VTR: 30 g/m/24 h. VP—forming: 175 μm nylon/EVOH/enhanced polyethylene coextrusion. OTR: 0.4 ML/m/24 h. VTR: 3.3 g/m/24 h. VP—non-forming: 65 μm nylon/polyolefin plastomer coextrusion. OTR: 53.0 mL/m/24 h. VTR: 6.5 g/m/24 h. VSP—forming: 350 μm polyester/EVOH/polyethylene coextrusion. OTR: 1.8 mL/m/24 h. VTR: 2.8 g/m/24 h. VSP—non-forming: 127 μm PE/EVOH/LLDPE. OTR: 0.8 mL/m/24 h. VTR: 0.4 g/m/24 h.

**Figure 9:**
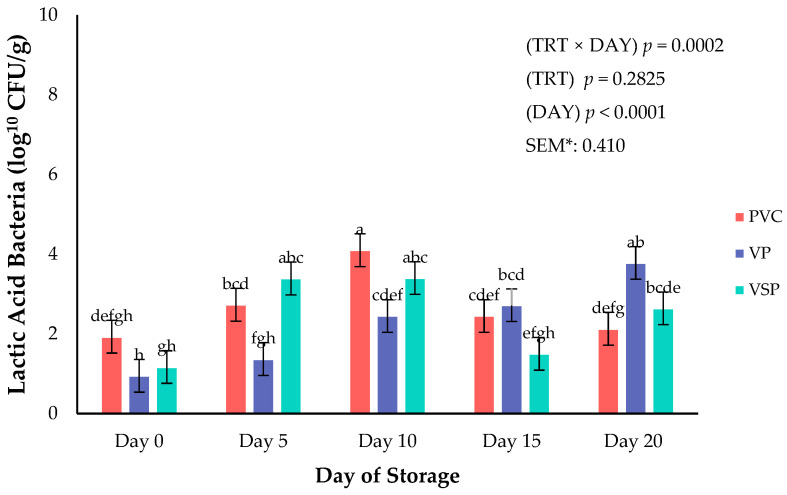
Interaction of storage duration (day) × packaging treatment on lactic acid bacteria growth. ^a–h^ Means lacking common superscripts differ (*p* < 0.05). * SEM, standard error of the mean. Packaging treatment (N = 7/Treatment/Day): PVC—10 μm polyvinyl chloride. OTR: 14,000 mL/m/24 h. VTR: 30 g/m/24 h. VP—forming: 175 μ nylon/EVOH/enhanced polyethylene coextrusion. OTR: 0.4 ML/m/24 h. VTR: 3.3 g/m/24 h. VP—non-forming: 65 μm nylon/polyolefin plastomer coextrusion. OTR: 53.0 mL/m/24 h VTR: 6.5 g/m/24 h. VSP—forming: 350 μm polyester/EVOH/polyethylene coextrusion. OTR: 1.8 mL/m/24 h. VTR: 2.8 g/m/24 h. VSP—non-forming: 127 μm PE/EVOH/LLDPE. OTR: 0.8 mL/m/24 h. VTR: 0.4 g/m/24 h.

Microbial growth for anaerobic (ANA), aerobic plate count (APC), and lactic acid bacteria (LAC) were assessed across all packaging types and throughout 20 days of storage duration. Significant interactions between packaging and day of storage were observed for microbial APC (*p* < 0.0001; Figure 7), ANA (*p* < 0.0001; Figure 8), and LAC (*p* = 0.0002; Figure 9) during the current study. Aerobic plate count on chicken in PVC and VSP packaging exhibited a greater increase by day 10, whereas chicken stored in VP had a slower aerobic organism growth. Specifically, aerobic growth did not differ regardless of packaging method but did not exceed a project threshold of 7-log. Spoilage threshold for the current project of microbial organisms was set at a limit of 7-log CFU/g as supported by historical research on fresh chicken deterioration [37]. It is well known throughout the literature that vacuum packaging effectively limits aerobic microbial growth due to the oxygen barrier properties of the packaging film [27,36,38]. Current results agree with previous research reporting vacuum environments will limit aerobic spoilage organisms [19,20,24,25]. Anaerobic populations were greatest on day 15 for all packaging types. However, by storage day 20 the number of ANA organisms was greatest for VP and packages of fresh chicken, indicating that facultative and anerobic populations can persist under vacuum and in agreement with previous reported results [39]. It is likely that the oxygen transmission rate of the packaging films leads to these changes in anaerobic organism growth during storage conditions. Lactic acid bacteria populations varied throughout storage time. LAC peaked on day 10 before declining and increasing again on day 20. This fluctuation could be due to the competitive interactions with other spoilage organisms or environmental changes during storage. Importantly, microbial growth across packaging and storage duration did not exceed the 7-log CFU/g spoilage threshold [37].

## 4. Conclusions

Vacuum-based packaging systems effectively extend the storage life of fresh poultry by preserving surface color, limiting microbial growth, and reducing lipid oxidation. In this study, packaging with low oxygen transmission rates, such as vacuum and vacuum skin formats, outperformed traditional PVC overwrap in slowing spoilage-related changes. However, despite improved microbial and oxidative stability, odor evaluation scores reached unacceptable levels by day 14 of storage. Volatile analysis confirmed the consistent presence of dimethyl sulfide, a sulfur-containing compound strongly associated with undesirable, cabbage-like odors, across all packaging types. These findings underscore a critical limitation: while vacuum packaging enhances shelf-life from a microbial and oxidative perspective, the point of sensory rejection by consumers—driven largely by odor—ultimately defines the usable shelf-life. Addressing this challenge will require targeted strategies to suppress or neutralize volatile sulfur compounds—through alternative packaging technologies, antimicrobial and antioxidant interventions, or real-time spoilage monitoring tools. Continued research in these areas will be essential to fully realize the commercial potential of vacuum-packaged poultry products.

## Figures and Tables

**Table 1:** Packaging film specifications used during storage of fresh boneless–skinless chicken breast.

Packaging Treatment ^1^	Film Type	Thickness	OTR ^2^	VPR ^3^
PVC	-	10 μm	14,000 mL/m/24 h	30 g/m/24 h
VP	Forming	175 μm	0.4 mL/m/24 h	3.3 g/m/24 h
	Non-Forming	65 μm	53.0 mL/m/24 h	6.5 g/m/24 h
VSP	Forming	350 μm	1.8 mL/m/24 h	2.8 g/m/24 h
	Non-Forming	127 μm	0.8 mL/m/24 h	0.4 g/m/24 h

^1^ Packaging treatment: PVC (polyvinyl chloride film); VP (thermoforming vacuum packaging); VSP (vacuum skin packaging). ^2^ OTR: oxygen transmission rate. ^3^ VPR: vapor transmission rate.

**Table 2:** Impact of packaging treatment on surface color of boneless–skinless chicken breast.

	Packaging Treatment				
	PVC	VP	VSP	SEM*	*p*-Value
REDNESS (a*)	5.58 ^a^	3.2 ^b^	2.63 ^c^	0.339	<0.0001
HUE ANGLE (°)	71.54	76.02	72.97	1.755	0.1749

Redness (a*)—a larger value indicates a redder color, where +60 is red and −60 is green. Hue angle (°)—change in color from the true red axis (a larger number indicates a greater shift from red to yellow). Packaging treatment (N = 10/Treatment): PVC—10 μm polyvinyl chloride. OTR: 14,000 mL/m/24 h. VTR: 30 g/m/24 h. VP—forming: 175 μm nylon/EVOH/enhanced polyethylene coextrusion. OTR: 0.4 mL/m/24 h. VTR: 3.3 g/m/24 h. VP—non-forming: 65 μm nylon/polyolefin plastomer coextrusion. OTR: 53.0 mL/m/24 h. VTR: 6.5 g/m/24 h. VSP—forming: 350 μm polyester/EVOH/polyethylene coextrusion. OTR: 1.8 mL/m/24 h. VTR: 2.8 g/m/24 h. VSP—non-forming: 127 μm PE/EVOH/LLDPE. OTR: 0.8 mL/m/24 h. VTR: 0.4 g/m/24 h. ^a–c^ Mean values within the main effect of day lacking common superscripts differ (*p* < 0.05). SEM*—standard error of the mean for day of storage.

**Table 3:** Impact of storage duration on surface color of boneless–skinless chicken breast.

	Day of Storage						
	0	5	10	15	20	SEM*	*p*-Value
REDNESS (a*)	3.14 ^b^	3.88 ^a^	3.99 ^a^	4.08 ^a^	3.93 ^a^	0.567	0.0100
HUE ANGLE (°)	72.89	71.55	75.04	74.52	73.55	2.266	0.8282

Redness (a*)—a larger value indicates a redder color, where +60 is red and −60 is green. Hue angle (°)—change in color from the true red axis (a larger number indicates a greater shift from red to yellow). ^a,b^ Mean values within the main effect of day lacking common superscripts differ (*p* < 0.05). SEM*—standard error of the mean for day of storage.

**Table 4:** Packaging influence on e-nose peak area volatile compounds in boneless–skinless chicken breast.

	Packaging Treatment				
Compound	PVC	VP	VSP	*p*-Value	SEM*
Aldehyde	295.98 ^b^	146.94 ^b^	2834.00 ^a^	0.0025	598.45
Alkane	655.23 ^a^	116.80 ^b^	505.95 ^b^	0.0143	162.82
Hydrocarbon	3925.89	2238.29	1141.75	0.7236	4256.64
Sulfur	607.20	440.22	302.80	0.4649	139.27
Furan	622.28	465.36	344.79	0.7630	285.81
Ketone	2653.01	2309.69	2075.15	0.5836	577.86
Terpene	239.93 ^a^	113.60 ^b^	47.91 ^b^	0.0214	44.47
Ester	3327.50	1944.81	1296.93	0.6280	1761.19
Carboxylic	154.35	85.61	128.67	0.1708	27.71
Alcohol	408.62	259.51	410.49	0.3475	83.53

^a,b^ Means lacking common superscripts differ (*p* < 0.05). * SEM, standard error of the mean. Packaging treatment (N = 2/Treatment): PVC—10 μm polyvinyl chloride. OTR: 14,000 mL/m/24 h. VTR: 30 g/m/24 h. VP—forming: 175 μm nylon/EVOH/enhanced polyethylene coextrusion. OTR: 0.4 ML/m/24 h. VTR: 3.3 g/m/24 h. VP—non-forming: 65 μm nylon/polyolefin plastomer coextrusion. OTR: 53.0 mL/m/24 h. VTR: 6.5 g/m/24 h. VSP—forming: 350 μm polyester/EVOH/polyethylene coextrusion. OTR: 1.8 mL/m/24 h. VTR: 2.8 g/m/24 h. VSP—non-forming: 127 μm PE/EVOH/LLDPE. OTR: 0.8 mL/m/24 h. VTR: 0.4 g/m/24 h.

**Table 5:** Impact of storage duration on e-nose peak area volatile compounds in boneless–skinless chicken breast.

	Day of Storage						
Compound	0	5	10	15	20	*p*-Value	SEM*
Alkane	100.92 ^b^	220.86 ^b^	368.03 ^b^	519.77 ^a^	920.39 ^a^	0.0118	206.29
Hydrocarbon	ND ^1^	ND ^1^	ND ^1^	452.80	4417.81	0.2275	2210.59
Sulfur	148.53 ^b^	65.66 ^b^	104.90 ^b^	568.75 ^b^	1362.53 ^a^	0.0040	299.11
Furan	123.71	ND ^1^	ND ^1^	949.05	359.68	0.1985	477.48
Ketone	1575.53	2544.94	2069.32	2568.81	2971.15	0.3198	517.44
Terpene	67.75	83.16	118.97	166.51	232.68	0.0941	133.81
Ester	3567.95	672.38	1125.81	3525.33	2057.27	0.8118	2319.44
Carboxylic	87.84 ^a^	60.16 ^a^	108.67 ^a^	234.84 ^b^	ND^1^	0.0008	55.07
Alcohol	223.91	413.99	350.14	306.65	503.13	0.3167	109.01

^a,b^ Means lacking common superscripts differ (*p* < 0.05). * SEM, standard error of the mean. ^1^ ND: Not detected.

**Table 6:** Volatile compound group mean retention time and relevance index by day of storage.

	Day of Storage										
Compound Group	Day 0		Day 5		Day 10		Day 15		Day 20		Sensory Descriptors ^3^
	RT ^1^	RI ^2^	RT ^1^	RI ^2^	RT ^1^	RI ^2^	RT ^1^	RI ^2^	RT ^1^	RI ^2^	
Alkane	63.57	91.74	49.40	92.20	46.39	88.59	50.61	91.04	52.04	91.00	Meaty
Hydrocarbon	ND ^4^	ND ^4^	ND ^4^	ND ^4^	ND ^4^	ND ^4^	45.27	92.28	37.86	91.13	Aldehydic; fatty
Sulfur	60.61	90.57	45.73	84.93	24.52	90.18	27.92	92.64	24.96	92.06	Cabbage; putrid
Furan	73.22	90.14	59.87	94.71	ND ^4^	ND ^4^	36.00	90.91	40.74	94.97	Metallic
Ketone	49.97	94.04	26.98	91.56	30.34	91.91	34.29	92.49	33.93	91.14	Fruity
Terpene	69.57	94.52	65.02	95.06	65.00	86.47	63.31	92.53	66.72	88.18	Terpenic
Ester	52.80	94.15	36.55	94.60	33.87	94.16	39.71	93.51	36.47	91.62	Onion; pungent
Carboxylic	43.34	91.91	33.20	89.42	34.76	88.47	29.86	90.86	ND ^4^	ND ^4^	Sour; rancid
Alcohol	41.82	89.60	26.78	89.31	26.76	89.34	34.79	91.21	41.84	89.66	Pleasant; burnt

^1^ Retention time of compounds. ^2^ Relevance index of compounds. ^3^ Sensory descriptors from AlphaSoft (version 7.2.8, Toulouse, France) Software Library. ^4^ Compound group not detected.

**Table 7:** Mean volatile compound group retention time and relevance index by packaging treatment.

	Treatment						
Compound Group	PVC		VP		VSP		Sensory Descriptors ^3^
	RT ^1^	RI ^2^	RT ^1^	RI ^2^	RT ^1^	RI ^2^	
Aldehyde	35.91	93.13	35.18	91.39	36.13	91.56	Pungent
Alkane	52.64	90.85	56.12	91.46	53.74	90.31	Meaty
Hydrocarbon	44.76	94.21	60.38	90.43	36.43	91.08	Aldehydic; fatty
Sulfur	39.29	92.96	33.08	91.82	29.98	89.86	Cabbage; putrid
Furan	63.46	92.78	61.21	91.99	62.87	90.89	Metallic
Ketone	41.19	89.07	36.58	94.56	30.61	94.36	Fruity
Terpene	63.25	93.07	67.51	91.76	66.38	89.79	Terpenic
Ester	41.35	93.37	32.47	94.65	38.06	91.99	Onion; pungent
Carboxylic	44.04	90.43	29.60	88.45	27.19	91.72	Sour; rancid
Alcohol	38.87	89.47	36.19	88.69	31.99	91.63	Pleasant; burnt

^1^ Retention time of compounds. ^2^ Relevance index of compounds. ^3^ Sensory descriptors from AlphaSoft (version7.2.8, Toulouse, France) Software Library. Packaging treatment (N = 2/Treatment): PVC—10 μ polyvinyl chloride. OTR: 14,000 mL/m/24 h. VTR: 30 g/m/24 h. VP—forming: 175 μ nylon/EVOH/enhanced polyethylene coextrusion. OTR: 0.4 ML/m/24 h. VTR: 3.3 g/m/24 h. VP—non-forming: 65 μ nylon/polyolefin plastomer coextrusion. OTR: 53.0 mL/m/24 h. VTR: 6.5 g/m/24 h.; VSP—forming: 350 μ polyester/EVOH/polyethylene coextrusion. OTR: 1.8 mL/m/24 h. VTR: 2.8 g/m/24 h. VSP—non-forming: 127 μ PE/EVOH/LLDPE. OTR: 0.8 mL/m/24 h. VTR: 0.4 g/m/24 h.

## Data Availability

The original contributions presented in the study are included in the article, further inquiries can be directed to the corresponding author.

## Abbreviations

The following abbreviations are used in this manuscript:

PVC	Polyvinyl chloride
VP	Vacuum packaging
VSP	Vacuum skin packaging
OTR	Oxygen transmission rate
VTR	Vapor transmission
EVOH	Ethylene-vinyl alcohol co-polymer
TBARS	Thiobarbituric acid reactive substances
ANA	Anaerobic bacteria
APC	Aerobic plate count
LAB	Lactic acid bacteria
SEM	Standard error of the mean
